# Electrospun nanofibers synthesized from polymers incorporated with bioactive compounds for wound healing

**DOI:** 10.1186/s12951-024-02491-8

**Published:** 2024-04-27

**Authors:** Naveen Palani, Pradeshwaran Vijayakumar, P. Monisha, Saravanakumar Ayyadurai, Suriyaprakash Rajadesingu

**Affiliations:** 1 Department of Physics and Nanotechnology, SRM Institute of Science and Technology, Chengalpattu District, Kattankulathur, 603 203 Tamil Nadu India; 2 Department of Chemistry, SRM Institute of Science and Technology, Chengalpattu District, Kattankulathur, 603 203 Tamil Nadu India; 3 PG & Research Department of Physics, Sri Sarada College for Women, Salem, 636 016 Tamil Nadu India; 4 Centre for Research in Environment, Sustainability Advocacy and Climate CHange (REACH), Directorate of Research, SRM Institute of Science and Technology, Chengalpattu District, Kattankulathur, 603 203 Tamil Nadu India

**Keywords:** Wound dressing, Nanomaterial, Nanofibrous mats, Antibacterial, Electrospining

## Abstract

The development of innovative wound dressing materials is crucial for effective wound care. It’s an active area of research driven by a better understanding of chronic wound pathogenesis. Addressing wound care properly is a clinical challenge, but there is a growing demand for advancements in this field. The synergy of medicinal plants and nanotechnology offers a promising approach to expedite the healing process for both acute and chronic wounds by facilitating the appropriate progression through various healing phases. Metal nanoparticles play an increasingly pivotal role in promoting efficient wound healing and preventing secondary bacterial infections. Their small size and high surface area facilitate enhanced biological interaction and penetration at the wound site. Specifically designed for topical drug delivery, these nanoparticles enable the sustained release of therapeutic molecules, such as growth factors and antibiotics. This targeted approach ensures optimal cell-to-cell interactions, proliferation, and vascularization, fostering effective and controlled wound healing. Nanoscale scaffolds have significant attention due to their attractive properties, including delivery capacity, high porosity and high surface area. They mimic the Extracellular matrix (ECM) and hence biocompatible. In response to the alarming rise of antibiotic-resistant, biohybrid nanofibrous wound dressings are gradually replacing conventional antibiotic delivery systems. This emerging class of wound dressings comprises biopolymeric nanofibers with inherent antibacterial properties, nature-derived compounds, and biofunctional agents. Nanotechnology, diminutive nanomaterials, nanoscaffolds, nanofibers, and biomaterials are harnessed for targeted drug delivery aimed at wound healing. This review article discusses the effects of nanofibrous scaffolds loaded with nanoparticles on wound healing, including biological (in vivo and in vitro) and mechanical outcomes.

**Graphical Abstract:**

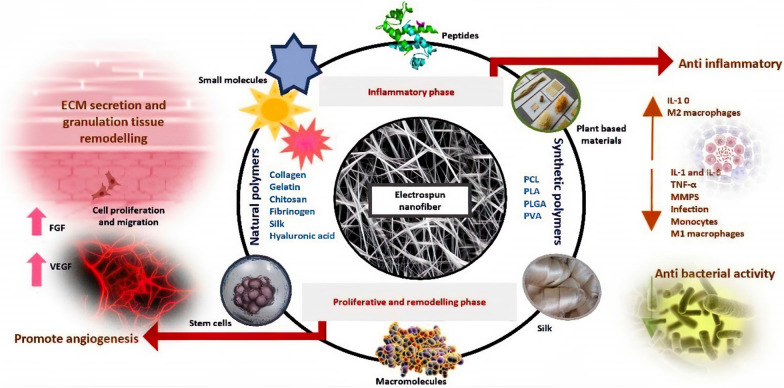

## Introduction

The skin is the body’s largest organ and acts as a protective barrier against various types of damages. It has two layers: the epidermis and the dermis. The epidermis consists of sensory axons, immune cells, and keratinocytes. The dermis is made up of skin appendages, fibroblasts, and mast cells. The goal of tissue engineering is to create a scaffold with biocompatibility, controlled biodegradability, and the right mechanical properties to repair damaged skin [1, 2]. During injury, it impacts the surface of our skin and the tissue beneath, forming a wound. Healing is a natural process that involves intricate mechanisms to restore the skin's normal structure and functions [3–5]. The skin has a variety of functions and acts as a protective barrier, shielding our bodies from harmful external factors like bacteria, UV radiation, and chemicals. The skin also helps regulate body temperature by sweating and dilating or constricting blood vessels. It allows us to sense touch, pressure, heat, and cold through specialized nerve endings. The skin plays a role in immune defense by housing immune cells that can detect and fight off pathogens. It also helps in the synthesis of vitamin D when exposed to sunlight [6]. The skin has developed a set of mechanisms called the wound-healing response to heal breaches quickly and effectively in its barrier [7]. According to recent research, the global market for wound care will increase from 19.8 billion USD in 2021 to up to 27.8 billion USD in 2026 at a CAGR of 7.6% [8]. Since the skin is increasingly susceptible to burns and injuries from operations or traumas. Several things can trigger wounds, including external injuries, genetic conditions, minored abrasions, burns, and surgical procedures [9]. Impaired healing can impact the recovery of various types of wounds, including both acute wounds (surgical incisions or traumatic injuries) and chronic wounds (diabetic foot ulcers). Acute wounds are minor cuts that usually heal within a few days [10]. As cells migrate, inflammation occurs, nerves grow, and new blood vessels form. Major surgeries can take a while to heal and may leave noticeable scars. Chronic wounds, which are persistent barrier defects that haven't healed in 3 months, can be challenging to treat [11]. To effectively manage wounds in modern therapy, it is crucial to ensure thorough cleaning, removal of foreign objects, relieving pressure, using a suitable dressing, and administering antibiotics if required [12].

**Fig. 1:**
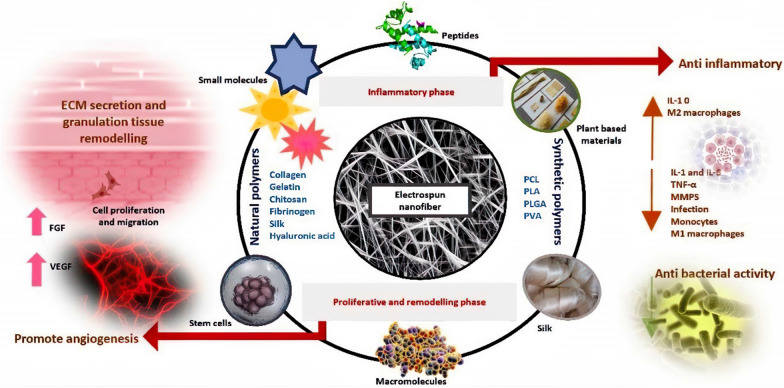
Schematic representation [21]

Traditional medicine has provided valuable insights into wound healing. Integrating nanotechnology into this field seems like a promising approach as shown in Fig.1. By incorporating bioactive compounds into nanomaterials, researchers can create innovative wound dressings and delivery systems that optimize tissue regeneration and minimize infection risks. The combination of ancient wisdom and modern science can lead to more effective and targeted interventions in wound healing. This interdisciplinary approach has the potential to greatly advance treatment outcomes. Since ancient times, the use of medicinal plant extracts has been a part of cultural heritage, especially in traditional medicine. These plant-based remedies, such as infusions, ointments, and compresses, have shown promising wound healing properties. This valuable knowledge has been passed down through generations and can be explored further in historical texts for pharmaceutical research [13]. Various therapies are used for wound management, surgical removal of necrotic tissue, and medication, but the results are still unsatisfactory. There is a need for new, affordable, and effective therapies in wound care. Researchers are now focusing on herbs used in folk medicines worldwide as potential therapeutic agents in wound healing [14]. Traditional medicine often touts the effectiveness of various medicinal plants for wound healing, but their scientific mechanisms and efficacy require further exploration. Wound healing is a well-coordinated process involving biochemical and cellular events, where damaged tissue attempts to repair itself. This intricate process involves the interaction of blood cells, mediators, and growth factors, ultimately regenerating healthy tissues and skin [15]. Plants contain phytochemicals that have antioxidant properties and can aid in wound healing. When reactive oxygen species (ROS) levels are high, they can hinder the wound-healing process by damaging cellular membranes. Additionally, excessive ROS can cause significant tissue injuries and potentially even lead to neoplastic transformation [16].

Nanotechnology revolves around nanoparticles, with a three-dimensional structure and a size ranging from 1 to 100 nm. Nanoparticles can be synthesized in various sizes and shapes including rod, spherical and crystal forms. There are different methods used to create nanoparticles. (i) Chemical methods can harm the environment and living organisms. Scientists are actively exploring and discovering highly efficient and cost-effective methods that are safe and eco-friendly for producing nanoparticles. (ii) The biological method is gaining popularity in nanoparticle production due to its minimal toxicity and environmental impact [17]. Water is used as a solvent to synthesize the nanoparticles from plant extracts. This method is safe and helps eliminate the potential risks. Water is a versatile and widely available solvent for many applications [18]. The green synthesis approach for nanoparticles is eco-friendly, cost-effective, and time-efficient. In addition, nanoparticles could be coated with bioorganic composites that make them proper for the biological environment. Using systemic antibiotics for wound therapy can lead to unwanted side effects and antimicrobial resistance. However, using topical antimicrobial agents allows the use of a higher concentration of medication directly in the affected area, reducing the negative effects of systemic drug administration and preventing the occurrence of antimicrobial resistance [19]. Therefore, wound dressing applications utilize many biopolymers and bioactive compounds obtained from natural resources [20] (Fig. 1).

**Fig. 2:**
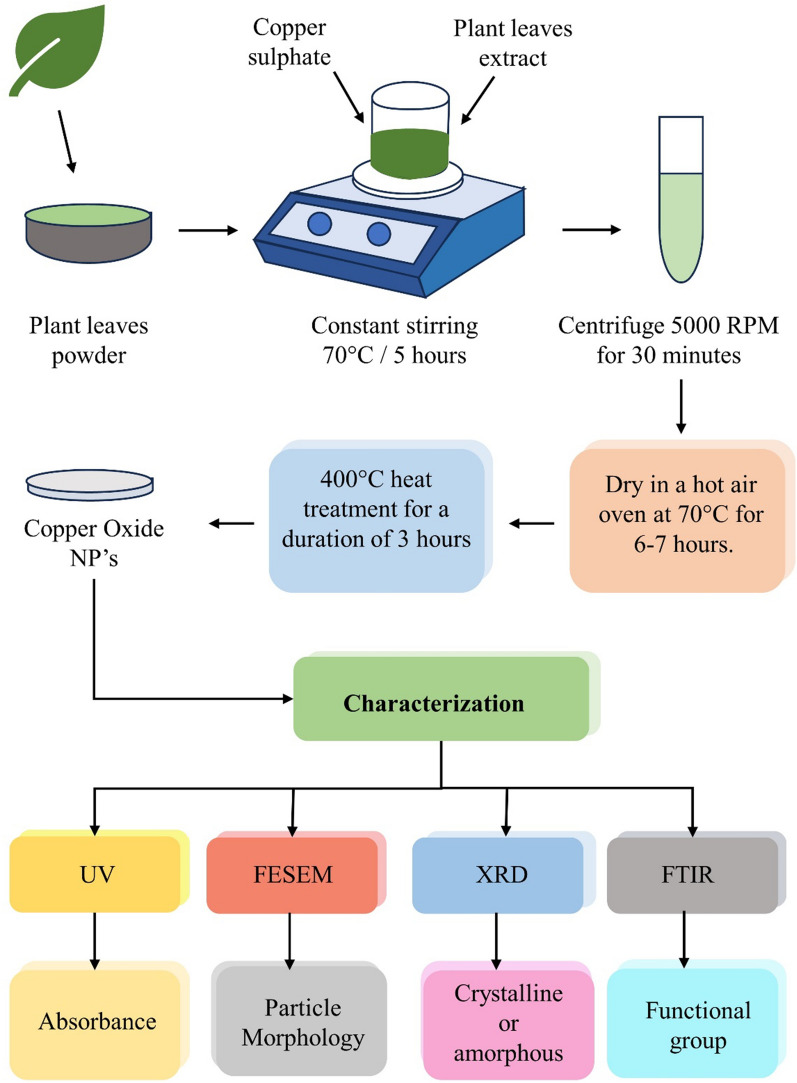
The process for synthesizing nanoparticles using an eco-friendly approach

Among the methods devised for the fabrication of nanofibers, electrospinning is one of the most popular techniques due to its cost-effectiveness and efficiency in producing ultrafine nanofibrous structures from diverse polymers. To increase the wound healing process, designing nanofibrous mat/wound dressing materials with antibiotic and antibacterial properties is essential [22]. Nanofibrous scaffolds have several advantages over conventional dressings (Fig. 2). They have a large surface area-to-volume ratio, tiny pore size, and high porosity. These properties allow for better exudate absorption improved wound permeation, and can help prevent further infection [23]. Electrospun nanofibers have the potential to revolutionize wound healing approaches. These nanofibers, made from both synthetic and natural polymers, can be incorporated with bioactive compounds to enhance wound healing outcomes. By exploring the synthesis techniques, material selection, and the synergistic effects of bioactive compounds, researchers can gain a comprehensive understanding of how to elevate wound healing.

## Process of wound healing

**Fig. 3:**
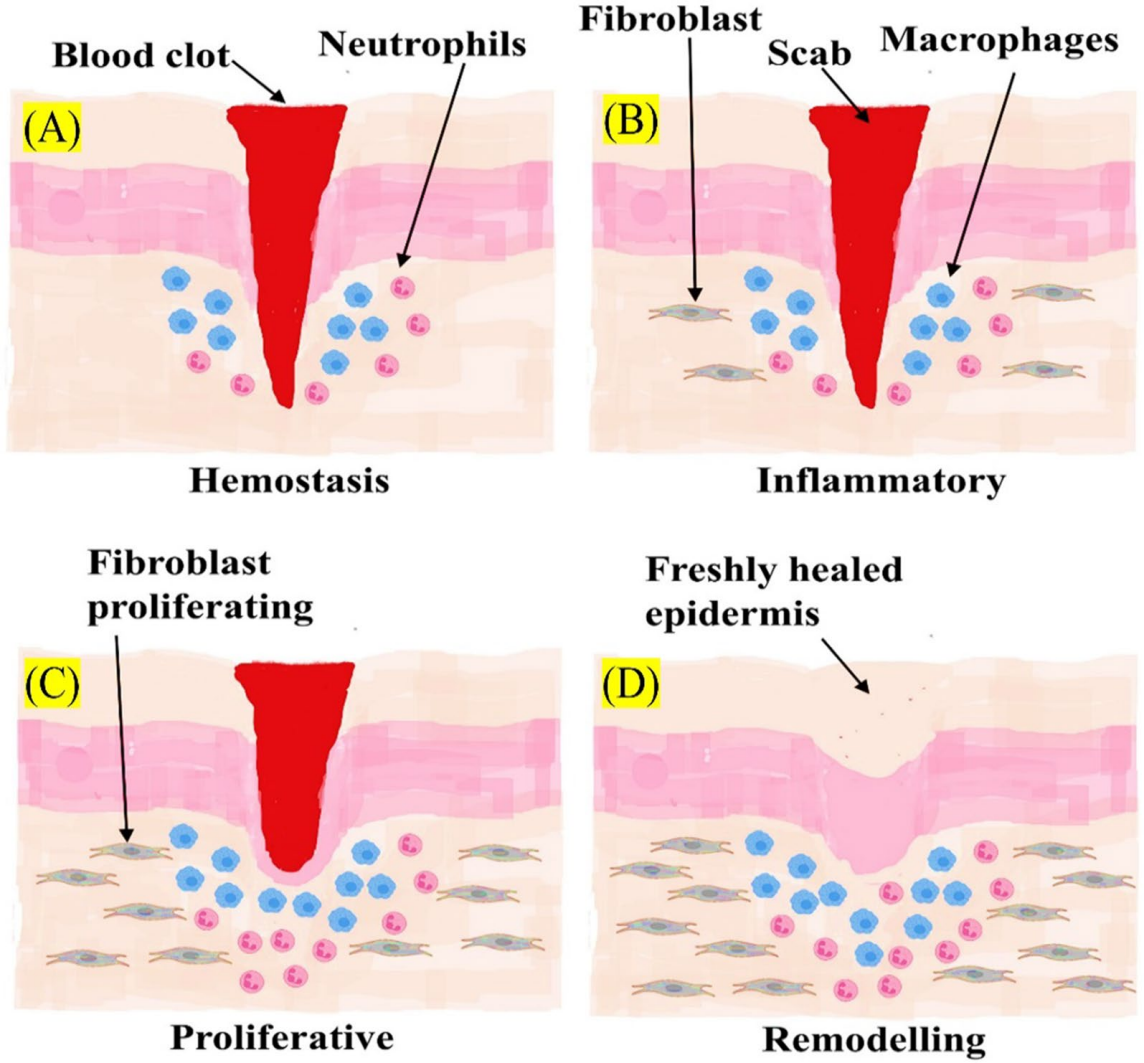
Stages of wound healing: **A** Hemostasis, **B** Inflammatory, **C** Proliferation, **D** Remodelling

There are four stages in the healing process: Hemostasis, inflammation, proliferation, and regeneration (Fig. 3**)** [24]. The process involves multiple growth factors, cytokines, and chemokines, forming a complex signalling mechanism. Cell proliferation plays a crucial role in tissue repair and regeneration during the healing process of wounds [25, 26].

### Hemostasis phase

In the hemostasis phase after skin damage, collagen triggers intrinsic and external clotting cascades, stopping the initial bleeding. Thrombocythemia aggregates and causes vasoconstriction, leading to hypokalemia, increased glycolysis, and changes in pH to minimize the blood loss. A blood clot forms to fill the wound bed, which serves as a temporary wound matrix and provides a scaffold for the migration of various cell types **(**Fig. 3A**)**. After 5 to 10 min of vasoconstriction, the blood vessels expand, and leukocytes and thrombocytes enter the posterior matrix [27, 28]. Additionally, several cytokines and growth factors are secreted within the wound during this stage to promote cell synergy and communication, allowing it to heal [29, 30].

### Inflammatory phase

**Fig. 4:**
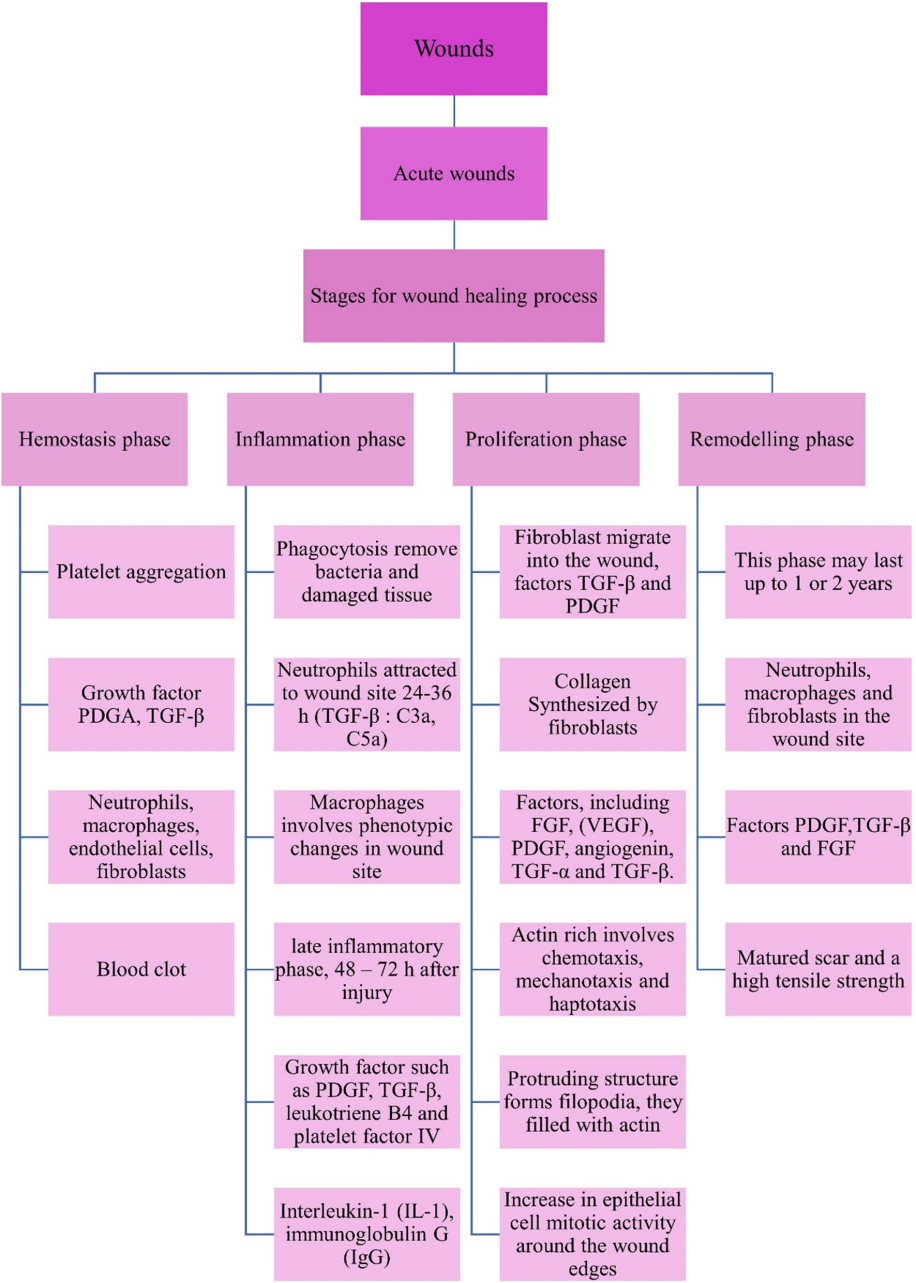
Stages of acute wound healing process

**Fig. 5:**
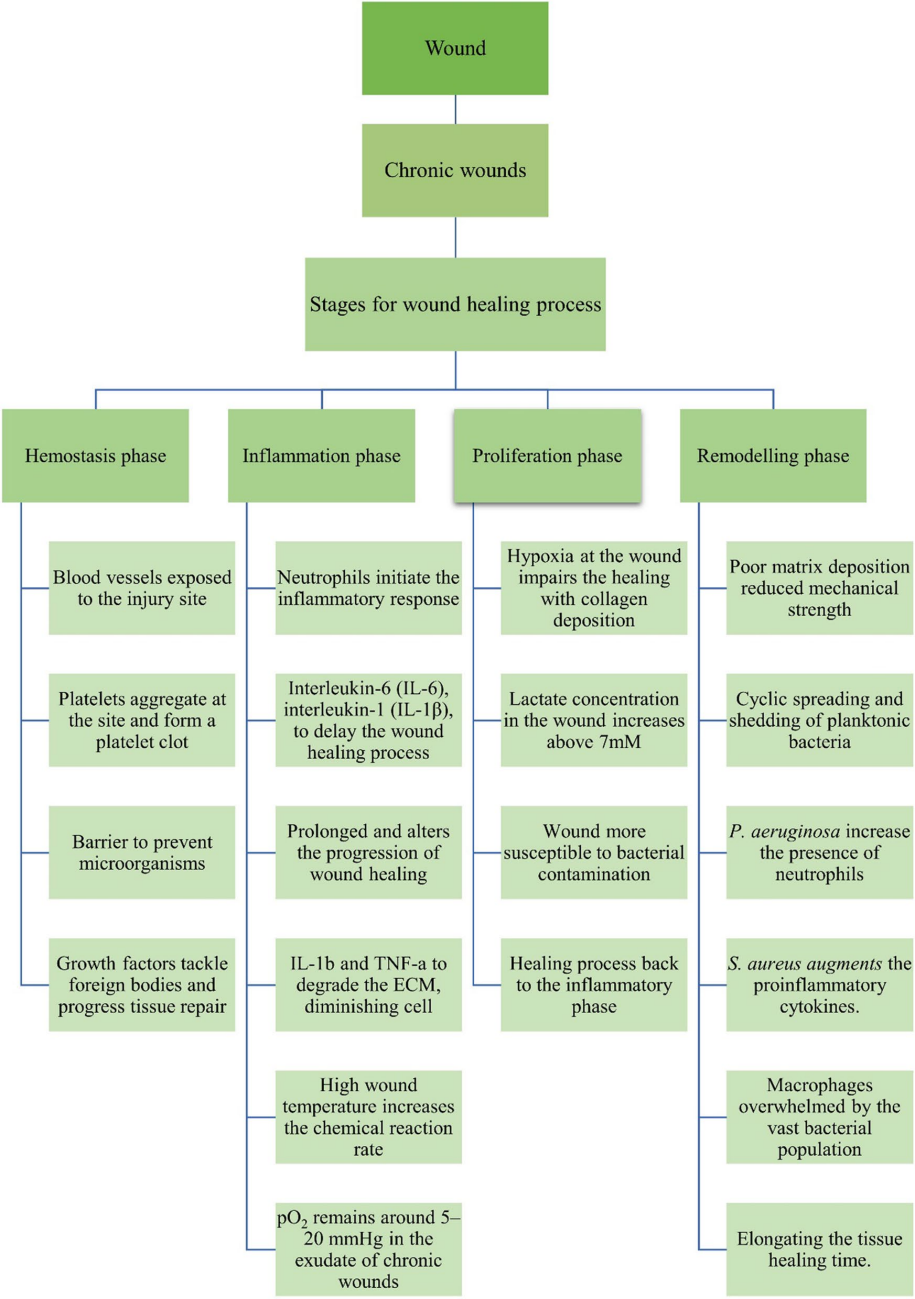
Factors Influencing Chronic wound healing process

The inflammatory phase destroys bacteria that have entered the wound during injury and provides the immune system with a barrier against bacterial contamination. The inflammatory response is divided into two phases: (i) The first stage of this process begins when a hemostatic response occurs, and white blood cells are observed at the injury site after being stimulated by hemostatic substances. (ii) In the second stage, a group of immune cells work together to kill bacteria through processes like phagocytosis, the production of reactive oxygen species, and proteinases for the removal of dead tissue. Neutrophils are key players in tissue removal. They stimulate and gather cells involved in the process (Fig. 3B). In parallel, their actions make it harder for extraterrestrials to survive. Many neutrophils transmigrate through endothelial cells in the blood capillary walls, which are activated by pro-inflammatory cytokines like tumor necrosis factor-alpha (TNF-α), interferon-gamma (IFN-γ), and IL-1, just a few hours after the lesion forms (Fig. 4). Neutrophils are either phagocytosed by macrophages, going through apoptosis, or emerging from the injury surface. Several growth factors, including platelet-derived growth factor (PDGF), transforming growth factor-beta (TGF-β), fibroblast growth factor (FGF), and vascular endothelial growth factor (VEGF), reach their highest concentrations in the wound two to three days after injury in macrophages, which are large phagocytic cells **(**Fig. 5**)**. These development factors are vital in controlling aggravation, animating angiogenesis, and arrangement of grind tissue [31].

### Proliferative phase

During this phase, neovascularization, and re-epithelialization take place. That process may take a few weeks to complete. Angiogenesis, in which new blood vessels are made from existing arteries, and vasculogenesis, in which endothelial progenitor cells (EPCs) make new arteries, play a role in neovascularization. Neovascularization re-establishes the tissue's access to nutrients. The process of gradually reducing the size of a wound and removing keratinocytes from its periphery is known as epithelialization. Initially, a shallow, thin layer of epithelial cells forms. A layer of stronger and thicker cells will eventually cover the wound over time. Anti-inflammatory mediators that promote collagen deposition, fibroblast proliferation, and angiogenesis are secreted by macrophages when they acquire the M2 phenotype [32, 33].

The wound expands once the collagen fibers replace the temporary fibrin mixture. Revascularization of the wound proceeds in the same direction as fibroplasia. Angiogenesis is the result of proliferation and migration working together (Fig. 3C). Capillary buds emerge from nearby blood vessels and spread into the wound space, aiding in the healing process. The process of angiogenesis encourages endothelial cells to migrate from the side of vessels that are close to the wound. Endothelial cell growth and chemotaxis are mediated by cytokines produced by platelets, macrophages, and lymphocytes in the wound, low oxygen tension, lactic acid, and biogenic amines (Fig. 5) [34]. Two principle reasons animate the fresh blood vessel arrangement, one is the microenvironment, including low oxygen strain, low pH, and high lactate levels; Another is the FGF, TGF- β, and VEGF, which aid in the EC's integrin-mediated connection to the Extracellular matrix (ECM) and facilitate healing (Fig. 4) [35].

### Remodelling phase

Redesigning is the last phase of twisted recuperating, during which the primary collagen organization of solid tissue is re-established. By synthesizing matrix metalloproteinases and new extracellular matrix, fibroblasts would control the decomposition of wound matrix, which promotes wound contraction, reduces epithelialization, and prevents scar formation (Fig. 4) [36]. In the healing process, the granulation tissue is gradually replaced by a more organized extracellular matrix, forming scar tissue with mechanical properties comparable to the tissue prior to the injury. Additionally, the blood vessel density in the granulation tissue returns to the level observed in healthy skin. Fibroblasts play a crucial role in the remodelling of collagen in the granulation tissue. They secrete matrix metalloproteinases that break down the collagen, allowing the formation of a more durable structure. The arrangement of the expressed collagens directly influences the process of wound healing (Fig. 3D). When wounds heal completely, the ratio of collagen type I to collagen type III increases, eventually matching that of normal skin. Over time, the collagen fibrils also increase in size, resembling a healthy dermis. Scar tissue, on the other hand, is predominantly composed of parallel bundles of collagen type I, which is weaker and less flexible compared to healthy skin tissue [37].

## Medicinal plants

Plants are rich in compounds like triterpenes, flavonoids, and alkaloids that can enhance wound healing. They can help with coagulation, fibroplasia, inflammation, collagenation, wound contraction, and epithelialization [38]. Medicinal plants offer a significant alternative in therapeutics, especially while dealing with multidrug-resistant microbial pathogens. Their phytochemical components, including antioxidants like flavonoids, polyphenols, and vitamins, have remarkable medicinal and therapeutic properties (Table 1). These antioxidants help prevent oxidative stress caused by reactive oxygen species and free radicals [39]. The mucilaginous gel extracted from the plant leaves has been utilized for its wound-healing properties, including antibacterial effects, and promoting collagen synthesis. It works by blocking the production of reactive oxygen species, prostaglandins, and cytokines while stimulating the growth of fibroblasts and keratinocytes. Carbohydrates in the gel activate immune cells like macrophages, which play a role in inflammation by releasing inflammatory mediators and engulf foreign particles or damaged cells. Studies have shown that aloe vera formulations, such as creams, gels, and impregnated dressings, have improved wound healing in both acute and chronic wounds in animal models [40, 41].

**Table 1:** Effects of medicinal plants on wound healing

Medicinal plants	Effects/activities	References
*Curcuma longa L*	Antimicrobial, tissue regeneration	[42, 43]
*Peucedanum ostruthium*	Antioxidant, anti-inflammatory, tissue growth	[44, 45]
*Abelmoschus esculentus L*	Antioxidant, anticancer, antibacterial	[46, 47]
*Semecarpus anacardium L* *Glochidion lanceolarium* *Bridelia retusa*	Antibacterial, anti-biofilm, antioxidant	[48]
*Tinospora cordifolia*	Antidiabetic, antioxidant, antimicrobial, anti-toxic effects, antistress activity, hypolipidemic effect, hepatic disorder, anti-HIV potential, anti-osteoporotic effects, anticancer	[49, 50]
*Rubia cordifolia* *Vetiveria zizanioides L* *Coscinium fenestratu L*	Antioxidant, increased rate of epithelization, anti-inflammatory	[51, 52]
*Persea americana,* *Ageratum conyzoides,* *Mangifera indica*	Antidiabetic	[53, 54]
*Cassia obtusifolia L*	Antimicrobial, increased tensile strength	[55]
*Phyllanthus muellerianus*	Increased blood vessels, increased tensile strength	[56, 57]
*Rhus coriaria,* *Globularia arabica* *Malva Sylvestri*	Antidiabetic, antiproliferative	[58, 59]
*Peucedanum ostruthium L*	Anti-inflammatory, antioxidant, protease inhibitory activity	[60, 61]
*Artemisia annua L*	Angiogenesis, antioxidant, antimicrobial	[62, 63]
*Chamaemelum nobile L*	Antibacterial, anti-biofilm, anti-inflammatory, anti-adhesion, tissue regeneration	[64, 65]
*Verbascum sinaiticum L*	Antioxidant, increased tensile strength	[66, 67]
*Antirrhinum majus*	Anti-biofilm, antimicrobial, antioxidant	[68, 69]
*Alternanthera sessilis*	Anti-inflammatory	[70, 71]
*Carica papaya*	Anti-diabetic, antioxidant, antimicrobial	[72, 73]

Flavonoids are found in different parts of plants, like seeds, bark, roots, and buds. Plants belonging to the family *Moraceae, Fabaceae, Guttiferae, Rutaceae, Euphorbiaceae*, and *Thymelaeaceae* consist of flavonoids. The leaves, fruits, and vegetables of *Artocarpus heterophyllus, Epimedium brevicornum, propolis,* and *Humulus lupulus* are used as medicines. Prenylated flavonoids have diverse structures, including C-prenylated chalcones, flavanones, flavonols, and isoflavones, with side chains like farnesyl, geranyl, and 3,3-dimethylallyl, 1,1-dimethylallyl [74]. These structures undergo modifications such as oxidation, reduction, dehydration, and cyclization. Prenylated flavonoids offer several benefits compared to regular flavonoids. They have a stronger attraction to the cell membrane at the desired location and exhibit powerful inhibitory properties against P-glycoprotein. These properties contribute to their ability to promote health [75]. There are more than 8000 diverse polyphenols, which can be divided into non-flavonoids and flavonoids. These plant-derived polyphenolic compounds have strong antioxidant activity, combating free radicals by providing a hydrogen atom or an electron. They also possess antimicrobial properties against specific bacteria in infected wounds and act as anti-inflammatory agents, reducing the inflammatory mediators linked to chronic wounds [76]. *Kigelia africana,* a medicinal plant, has been found to have beneficial effects in alleviating fungal infestations, psoriatic arthritis, dermatitis, and even cancer. Compounds like kigelinone, vernolic acid, kigelin, iridoids, luteolin, and 6-hydroxyluteolin in different plant parts contribute to its healing properties. In a study conducted by the Agyare research team in 2013, the methanolic extract of *Kigelia Africana* from the leaves and roots showed a 100% healing rate compared to the control, which had a healing rate of 96.59% [77]. *Dillenia indica* fruit extracts containing betulinic acid were tested in a laboratory to assess their ability to protect against lipid peroxidation [78]. In an animal study using rats with psoriasis-like wounds, the extracts with a 50 mg/mL concentration in betulinic acid promoted faster wound healing, reduced immune cell infiltration and parakeratosis, and exhibited anti-inflammatory effects. Another research study investigated the anti-inflammatory effects of betulinic acid from *Diospyros kaki* in macrophages stimulated by lipopolysaccharide [79].

A significant amount of alkaloids in *A. africana* is believed to be a critical factor in its wound-healing properties. [80] Various alkaloids have been known for their effective wound-healing effects, as demonstrated in studies using alkaloid-enriched ointments on rats. Additionally, alkaloids have shown the ability to promote early stages of wound healing by stimulating fibroblast chemotaxis [81]. The wound-healing potential of *A. africana* is likely attributed to its abundant alkaloid content [82]. The study compared the effectiveness of *Nigella sativa* oil and *Aloe vera* gel in diabetic foot wound healing. Aloe treatment showed improved wound area resolution and re-epithelialization compared to the oil and control [83]. Another study assessed an aqueous aloe extract for skin wounds in mice, showing improvements in epithelization and some mutagenic and cytotoxic effects [84]. Additionally, a survey of a porous wound dressing with aloe extract found it promoted cell growth and attenuated scar-forming myofibroblast formation. However, a study on *A. vera* extract for open wounds in rats found no significant difference compared to the control group [85]. The research discovered that the leaf extract of *Plumbago zeylanica L.* exhibited the highest antimicrobial activity against *Staphylococcus aureus*, while the stem extract was more effective against *Pseudomonas aeruginosa* [86]. Another study investigated the antibacterial properties of ethanolic extract from the root bark of Plumbago *zeylanica L.* The findings showed that the antibacterial activity increased as the concentration of the extract increased. [87]. In a recent experiment, *Plumbago zeylanica L*. also had excellent inhibitory activity against *Alternaria spp* and relatively less activity against *Sclerotium rolfsii* [88].

## Nanoparticle for a therapeutic drug

**Fig. 6:**
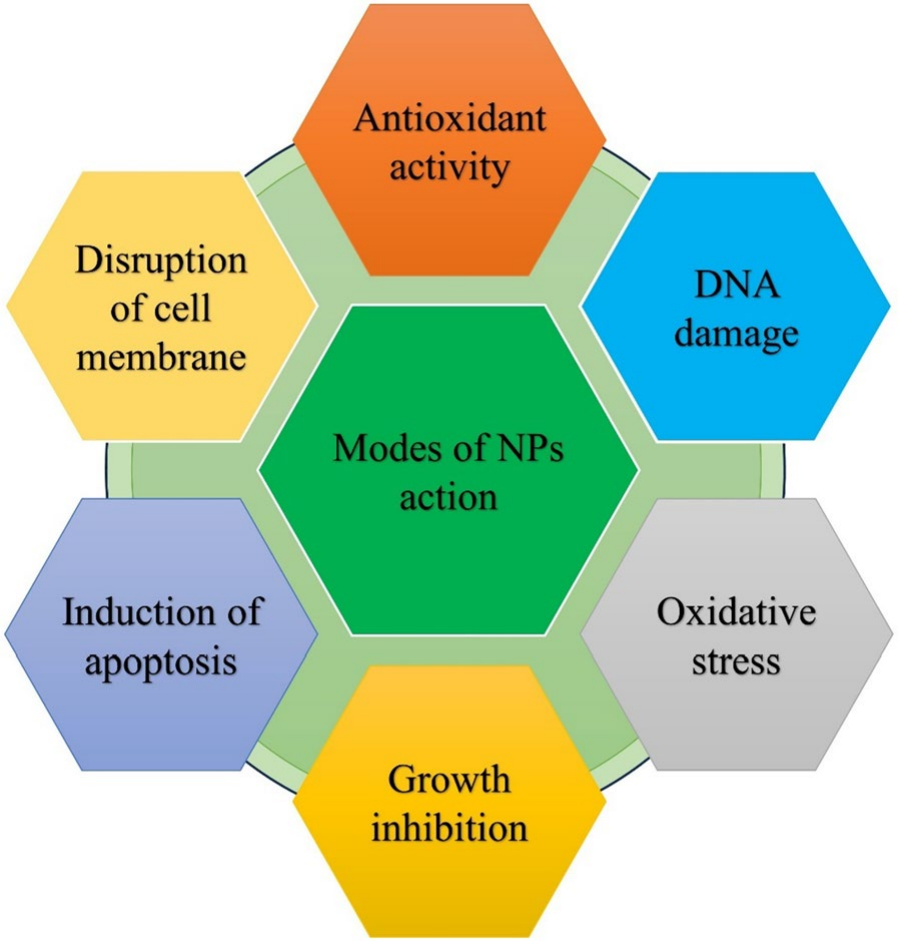
Different modes of nanoparticle action

Nanomaterials are beneficial to speeding up the injury recuperating process because of their profitable proportion of surface region to volume and medication conveyance ability. Collagen deposition and skin tissue regeneration may be affected by this feature. Mainly, the size of the nanoparticles enables them to penetrate the wound, allowing contact with specific target molecules and the local release of bioactive agents or drugs that influence the healing rate. Drugs are protected from wound-bed proteases by being encapsulated in nanocarriers, allowing them to carry out their biological function [89]. Nanoparticle modes refer to different ways in which nanoparticles can be utilized or interact with their surroundings. These modes can include things like surface modification, functionalization, encapsulation, or aggregation **(**Fig. 6**)**. It is widely recognized that providing therapeutic substances such as growth factors, antioxidants, antibiotics, and nucleic acids to affected tissue can significantly impact the healing of chronic wounds. These substances can stimulate cell growth, enhance cell movement, promote blood vessel formation, encourage collagen production, and combat microbial activity.

The nanoparticles and biomolecules can be added to hydrogels, leading to advanced topical drug delivery. This approach offers unique benefits like preventing sudden release, controlled sequential release, and improved targeting of specific tissues [90]. Over the past few decades, numerous nanomaterials with biological applications have received extensive research. Among these are Liposomes, dendrimers, quantum dots, fullerenes, carbon nanotubes, graphene, titanium oxide, iron, and gold nanoparticles. To encourage angiogenesis, NP-based delivery of ions like calcium and oxygen has been used [91]. NPs have become a crucial tool in various medical and pharmaceutical research areas. Their small size and customizable features make them highly effective for controlled and targeted drug release. In wound dressing development, NPs can be used as delivery vehicles or as bioactive components to enhance wound healing outcomes **(**Table 2**)**. They are excellent carriers for transporting active compounds to improve wound healing [92].

**Table 2:** Green nanoparticles enhancing wound healing

Nanoparticles	Plants	Reducing/capping agent	Properties	References
Ag	*Mimosa pudica* *Musa paradisiacal* *Eclipta prostrate*	Seed extract Flower extract	Highest rate of re-epithelialization	[93–97]
CeO_2_	*Zingiber officinale*	Leaves extract	Decrease the wound infection	[98–100]
GO	*Alovera*	Stem extract	Enhanced the angiogenesis	[101, 102]
CuO	*Caesalpinia bonducella*	Root extract Leaves extract	To resist microbial activity	[103, 104]
ZnO	*Ailanthus altissima* *Cinnamomum tamala Cinnamomum verum* *Brassica oleracea var*	Seed extract Leaves extract	Potential additive to substitute toxic chemical	[105–107]
Au	*Fusarium solani* *Anacardium occidentale*	Root extract	To help heal quickly to chronic wounds	[108–110]
SeO_2_	*Withania somnifera*	Leaves extract	Enhance antioxidant and antibacterial activities	[111]
Pd	*Anogeissus latifolia*	Leaves extract	Enhance antioxidant activities	[112, 113]
Fe_3_O_4_	*Couroupita guianensis*	Fruit extract	Reduce cytotoxicity activity	[114]
TiO_2_	*Trigonella foenum*	Leaves extract	Enhance antibacterial activities	[115]
CoFe_2_O_4_	*Abelmoschus esculentus*	Stem extract	Enhance antibacterial activities	[116]

### Zinc nanoparticles

**Fig. 7:**
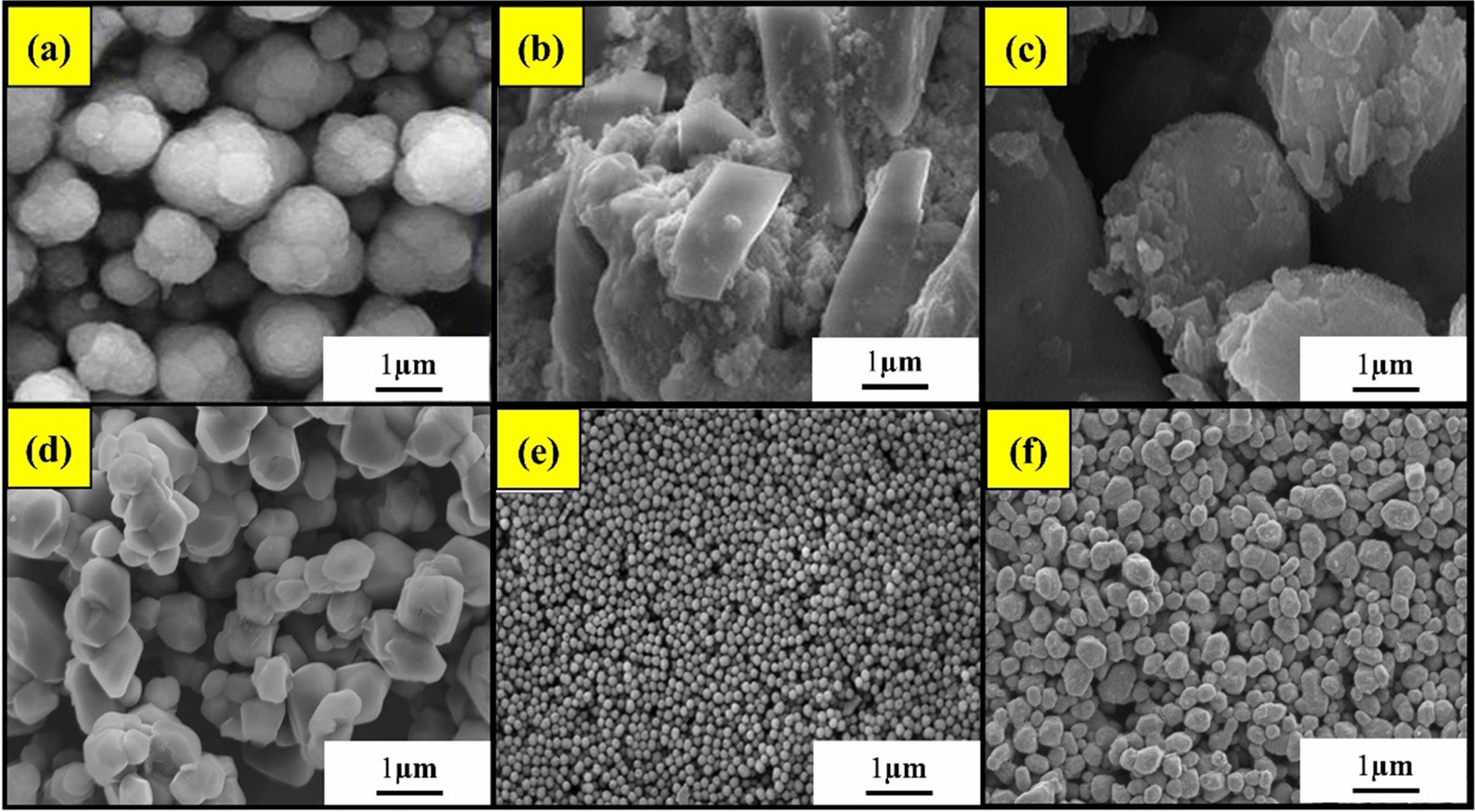
FE-SEM images of nanoparticles synthesized using a green approach 1 µm **a** ZnO NPs, **b** Au NPs, **c** Ag NPs, **d** CuO NPs, **e** CeO_2_ NPs, **f** TiO_2_ NPs.[143–148]

Zinc NPs are chemically stability, has antibacterial and anti-inflammatory properties, and release Zn^2+^ ions, which encourages keratinocyte migration during wound healing, ZnO NPs (Fig. 7a**)** used in wound dressings speed up the healing of acute and chronic wounds [117]. The carbohydrate-based polymer with ZnO NPs in chronic venous leg ulcers will enhance wound healing and anti-inflammatory effect [118]. When ZnO nanoparticles and cells come together, they form intercalated structures on the nanoparticle surface. These nanoparticles promote better cell adhesion and attachment, leading to high cell proliferation and growth. The extracellular matrix plays a role in cell attachment to the nanoparticles. Optimized ZnFe_2_O_4_ NPs show great potential in fighting drug-resistant bacteria and promoting wound healing. Nanoparticles are also being used in making bandages for burnt wounds. The potential of ZnS-NPs is demonstrated in both in vitro and in vivo studies as a possible therapy for skin regeneration. Before clinical application, further research is needed to validate these findings using advanced cell culture models and clinically relevant animal models. Overall, ZnS-NP shows promise in scarless wound repair [119]. Zinc ions released from ZnO have multiple beneficial effects on wound healing. They enhance keratinocyte migration toward the wound, promote healing, and exhibit antibacterial activity by interacting with bacterial cell membranes. Additionally, the topical application of ZnO-NPs stimulates angiogenesis re-epithelization and reduces inflammation and bacterial growth. These properties have been observed in equine wounds, leading to accelerated healing and clearance of visible signs of infection [120].

### Silver nanoparticles

Silver nanoparticles (AgNPs) possess unique properties that enable them to disrupt the microbial membrane, infiltrate the microbe's body, and cause internal damage. Their small size and large surface area contribute to their effectiveness in combating microbes. When bacteria come in contact with silver nanoparticles (Fig. 7c**)**, they tend to clump together near the bacterial membrane. Nanoparticles can lead to cell breakdown and lipopolysaccharide (LPS) removal through membrane protrusions that bind to the nanoparticles. The nanoparticles enter the cell through electrostatic attraction. Silver nanoparticles (Ag NPs) have antimicrobial properties due to their ability to induce oxidative stress, release metal ions, and employ other non-oxidative mechanisms [121]. Even after prolonged use, dressings containing Ag NP do not appear to inhibit the proliferation of fibroblasts and keratinocytes, restoring normal skin [122]. Late precise audits and meta-examinations report silver sulfadiazine to have more unfortunate recuperating results and little proof of viability in forestalling twisted contamination than substitute dressings. Components accepted to be answerable for these results are the prerequisite for customary injury dressing changes, unfortunate eschar infiltration, and cytotoxicity of silver to keratinocytes and fibroblasts, deferring wound recuperating [123, 124].

### Gold nanoparticles

Gold nanoparticles (AuNPs) have shown (Fig. 7b**)** promising results in viability studies with immune system cells, as they were found to be non-toxic and even reduced harmful reactive oxygen species [125]. Citrate-capped gold nanospheres were also found to be non-toxic in skin cells. AuNPs can easily permeate the skin, making them potential carriers for transdermal drug delivery without any toxic effects. In studies on rabbits and guinea pigs, AuNPs thermos responsive gels did not cause skin irritation or sensitization, making them a smart option for topical antibacterial drug delivery in cases of skin inflammation and wound healing [126]. The combination of nanogold, hyaluronic acid, and an adipocyte-targeting peptide has shown great promise in promoting lipolysis and breaking down fats. Irradiating the targeted site with a near-infrared laser selectively induces lipolysis in white fat, reducing body weight in obese mice. Additionally, injecting nanogold-coated polyethylene glycol into adipose tissue can enhance the effects of liposuction. In recent years, nanogold has also played a crucial role in developing coronavirus vaccines and virus detection, highlighting its significance in biomedicine [127]. The wound-healing efficiency of Au NRs was remarkable, after 14 days of daily treatment, they demonstrated almost completely healed wounds due to their enhanced skin re-epithelization effect and collagen formation. They also impacted the gene expression of inflammatory and anti-inflammatory mediators. Additionally, the deposition of Au NRs into different organs after topical wound treatment was insignificant. Gold nanoparticles made Au NRs-loaded into poloxamer 407 hydrogel, a promising gold-based nano-platform for accelerating wound healing [128].

### Copper nanoparticles

After a thorough toxicity evaluation, copper-based nanomaterials could be used to speed up wound healing (Fig. 7d). In rats, the growth of keratinocytes, blood vessel formation (angiogenesis), fibroblast activity, collagen production, and the process of skin cell regeneration were all boosted by copper nanoparticles and enhanced wound healing in diabetic mice by producing key signal proteins like TGF, MMP-2, and VEGF. Additionally, copper nanoparticle-based nanocomposites are important in guiding cytokines, cells, and growth factors that promote wound recovery. The wound healing in mice was sped up by copper nanocomposites made with chitosan. These nanocomposites increased the levels of interleukin-10 (IL-10), VEGF, and TGF-1, while reducing tumor necrosis factor (TNF) [129]. Copper has strong biocidal properties and is well-metabolized by the human body, unlike silver. It plays a crucial role in skin regeneration and angiogenesis, accelerating the healing process in animal models. Copper enhances the expression of HIF-1α, which induces VEGF and angiogenesis, promoting wound healing. HIF-1α also binds to essential regions in the promoter and enhancer of HIF-1-regulated genes, further aiding in wound healing induced by copper [130].

### Titanium dioxide

TiO2 NPs have remarkable antibacterial efficacy against a wide range of pathogens (Fig. 7f). They can inhibit bacterial growth through various mechanisms, making them a promising antimicrobial agent for wound dressings and coatings. TiO_2_ NPs also possess angiogenic properties, promoting blood vessel formation and facilitating efficient wound healing [131]. They have antioxidant effects as well, helping to scavenge reactive oxygen species and reduce oxidative stress in the wound microenvironment. Recent clinical studies have shown promising outcomes, and researchers are exploring different formulations like gels, creams, and dressings [132].

### Cerium oxide

CeO_2_ NPs have strong antibacterial efficacy and can inhibit bacterial growth and biofilm formation (Fig. 7e). This makes them a potential antimicrobial agent for wound dressings and coatings. It's fascinating that CeO_2_ NPs also have angiogenic properties, promoting neovascularization and improving blood flow and tissue regeneration in wounds [133]. They also exhibit antioxidant effects, helping to scavenge reactive oxygen species and reduce oxidative stress in the wound microenvironment. Tthe translational potential of CeO2 NPs in wound care, developing formulations like hydrogels or dressings [134, 135].

### Bioactive glass nanoparticles

Bioactive glass nanoparticles (BG NPs) have shown that they have impressive antibacterial properties, inhibiting microbial growth and preventing biofilm formation. This makes them a promising solution for combating infections in wounds [136, 137]. BG NPs have angiogenic properties, stimulating the formation of new blood vessels and promoting efficient nutrient and oxygen delivery to the wound site. This contributes to accelerated tissue regeneration. Additionally, BG NPs have antioxidant effects, reducing oxidative stress in the wound microenvironment by scavenging reactive oxygen species [138, 139].

### Carbon nanotubes

Carbon nanotube (CNTs) have antibacterial properties, keeping bacterial growth in check and preventing infections in wounds. CNTs also can stimulate the formation of new blood vessels, improving blood circulation and ensuring that nutrients reach the wound site for tissue regeneration. They have antioxidant properties, helping to reduce oxidative stress in the wound area and enhancing the wound healing process [140].

### Graphene-based nanomaterials

Graphene nanoparticles have emerged as a revolutionary component in the realm of wound healing, showcasing distinctive antibacterial, angiogenic, and antioxidant properties. Recent studies highlight the remarkable antibacterial efficacy of graphene nanoparticles, illustrating their ability to inhibit microbial growth and thwart infection in wound sites [141]. The angiogenic potential of graphene nanoparticles reveal their capacity to stimulate the formation of new blood vessels, promoting efficient nutrient and oxygen delivery critical for accelerated tissue regeneration. The multifaceted nature of graphene nanoparticles positions them as a promising and versatile tool for enhancing wound healing outcomes, signaling a transformative avenue in the landscape of advanced wound care [142].

## Electrospinning nanofiber technology

**Fig. 8:**
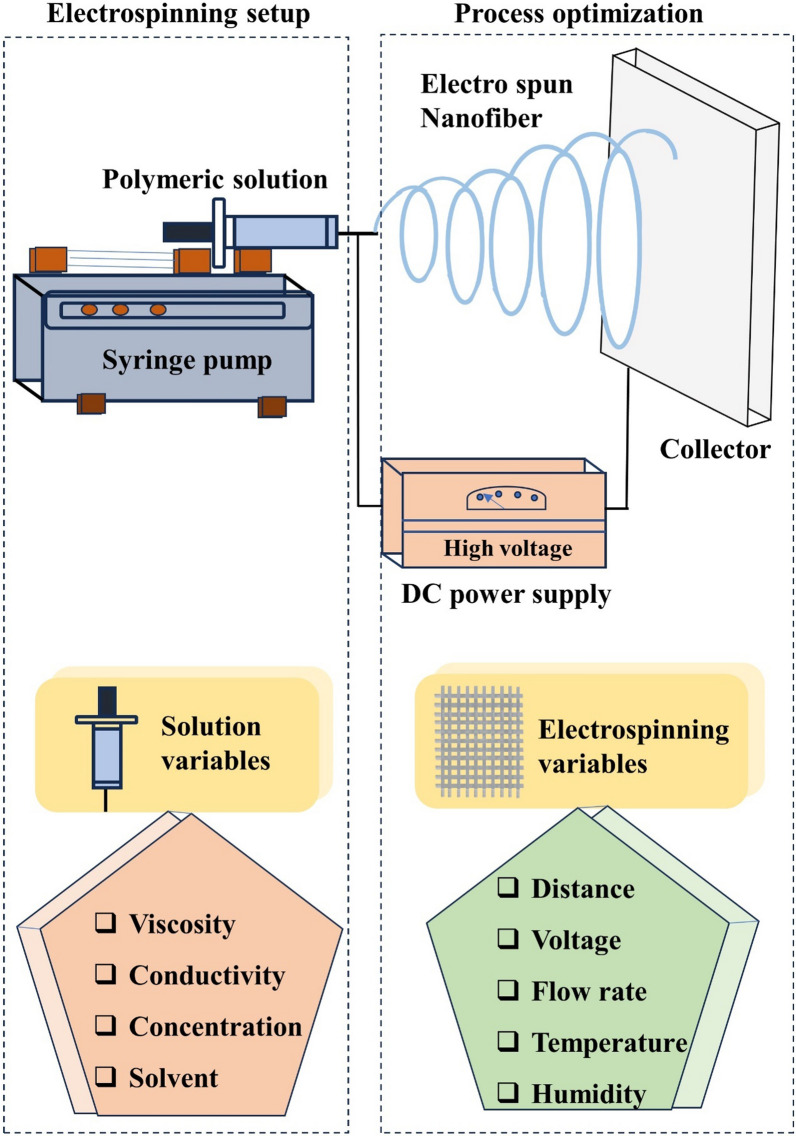
Schematic representation of electrospinning equipment and the optimization of polymer properties through adjustments in various parameters

Electrospinning is a technique that involves applying an electrostatic force to a polymer solution or melted polymer, resulting in the production of ultra-thin fibers. A high-voltage electrical supply, a needle/spinneret, and a grounded conductive collector are the main components of electrospinning (Fig. 8). The syringe is filled with the polymer solution, and the surface-electrified polymer solution holds fluid droplets. When the electrostatic force is applied, it creates repulsion between the like charges in the solution, causing the droplet to elongate into a cone shape called the “Taylor cone”. This cone forms a fiber jet as the repulsive forces overcome the surface tension. The jet stretches as it moves towards the collector. Eventually, the solvent evaporates, creating a solid non-woven fiber matt on the collector [149]. Electrospinning is a straightforward and adaptable method for extracting nanofibers (NFs) from viscoelastic fluids using electrostatic repulsion between surface charges. Because of their controllable diameter, arrangement, porosity, and surface properties as well as their ability to simulate extracellular matrix, electrospun nanofibers have been extensively utilized in tissue engineering. Electrospinning nanofibers are an ideal scaffold for skin tissue engineering because of their numerous wound-healing properties, high porosity, good hydrophilicity, controllable biodegradation, and good biocompatibility (Table 3) [150]. Electrospinning has been widely used to create nanofibrous materials that can be used as wound dressings because it is a process that is simple to operate, a wide variety of material options are available, and is economical. By tailoring dressings' physical and chemical properties, we can create highly specific dressings for wound healing applications. This customization allows for more effective and targeted treatment; synthetic and natural polymers are typically processed in blended solutions or co-electro-spun from different solutions [151]. Nanofiber wound dressings can prevent microbial infections by promoting high-gas permeation, supporting dermal drug delivery, enhancing fluid absorption, and stimulating hemostasis of damaged tissues and cell respiration. Nanofibers have incredible properties like enhanced mechanical strength, high porosity, and the ability to deliver bioactive substances. Electrospinning is an outstanding and highly effective method for producing polymeric nanofibers [152].

**Table 3:** Combination of polymers and plant compounds for wound-healing

Polymer	Ratio	Plant/Metabolites	Application	References
Polyethylene glycol/Polycaprolactone	2:1	*Naringenin*	Treat cutaneous wound infection	[186, 187]
Carboxymethylcellulose (CMC)	2:1	*Poincianella pluviosa*	Re-epithelialisation and enhanced skin organization	[188]
Chitosan/methacrylated silk fibroin	3:5:1	*Tannic acid*	Increase the strength of fibroblast cells	[189, 190]
Alginate/PVA	1:1,1:3 3:1	*Echinacea purpurea*	To treat dermal infection	[191]
Polycaprolactone/chitosan	2:1	*Hyaluronic acid*	To increase collagen fibrin and skin repair	[192]
Chitosan/gelatin	1:3	*oregano essential oil*	Healing of diabetic foot ulcers	[193–195]
Polyvinyl alcohol	1:1	*Euchima spinosum*	To increase the tensile strength	[196, 197]
Sodium alginate-g-poly (N-isopropyl acrylamide)	3:2	*Curcumin*	Enhanced collagenases and increased number of fibroblasts	[198, 199]
Polycaprolactone	3:1	*Plectranthus amboinicus* *Plantago major L*	Therapeutic material for skin wound healing	[200]
Chitosan/polyethylene oxide	1:2	*Calendula officinalis*	enhanced proliferation, growth and attachment of the cells	[201]
PVA/chitosan/silk fibrin	2:1:1	*Deferoxamine ciprofloxacin*	To treat the dermal fibroblast cells	[202, 203]

### Natural polymer

**Fig. 9:**
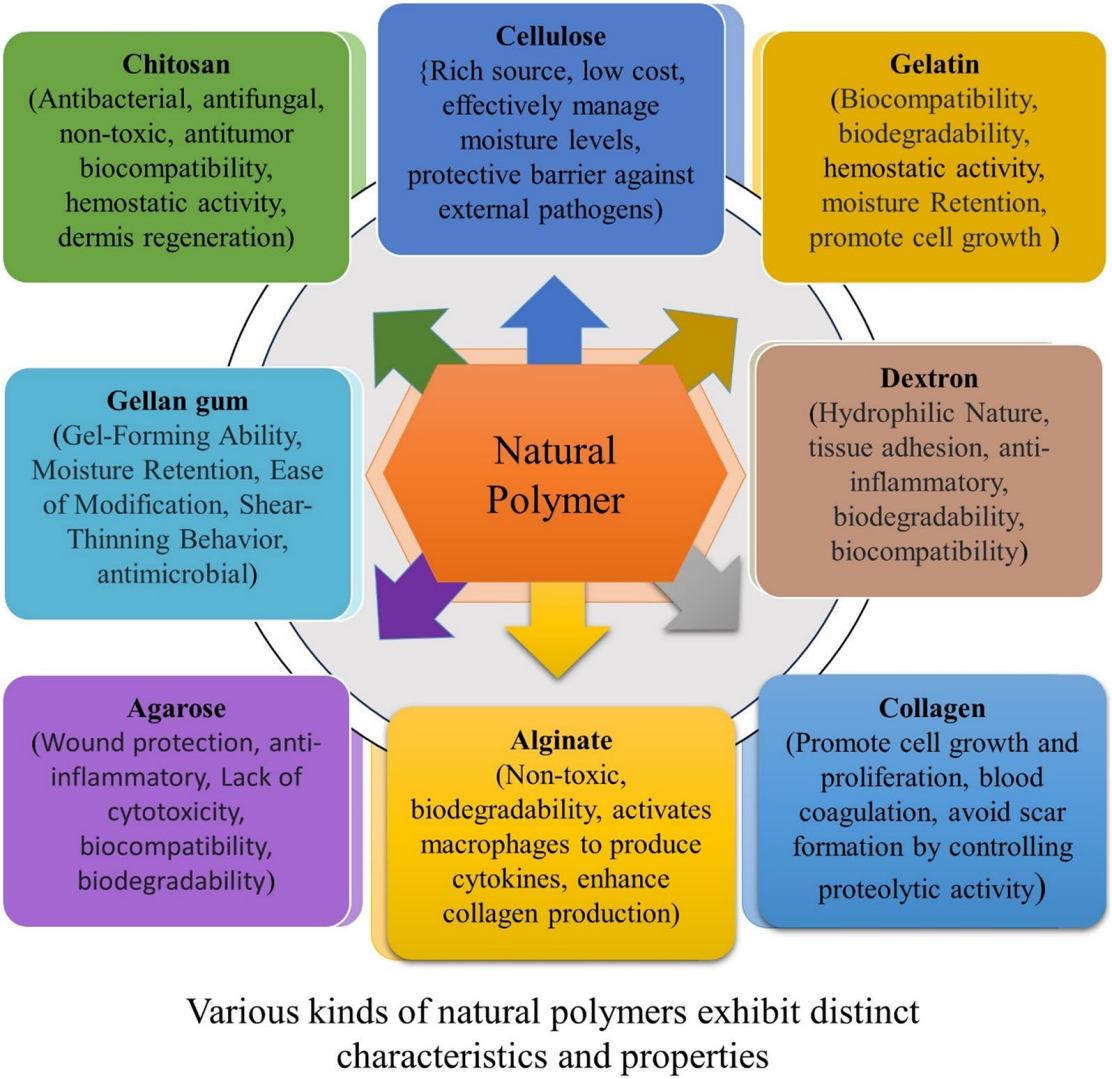
A natural polymer with a range of diverse polymer applications, each demonstrating unique and specific attributes related to wound healing activity

Natural polymers are often used in regenerative medicine for wound and burn dressings because they are biodegradable, biocompatible, and have a similar structure to the extracellular matrix. Prompting and animating the injury-mending process, regular polymers are engaged with the maintenance of harmed tissues and, subsequently, skin recovery. Radiation processing also produces novel biomaterials made from renewable, non-toxic, and biodegradable natural polymers (Fig. 9**)** [153]. Collagen and gelatin are commonly used in wound dressings and tissue engineering products because they have hemostatic properties, are biocompatible, have low cytotoxicity, and promote cellular attachment and growth [24]. It's incredible that there are over 3,000 different types of wound dressings available, catering to various aspects of wound care (Table 3).

#### Chitosan

The wound-healing properties of dressings made of chitosan that are used to repair wounds are quite intriguing. However, chitosan's low mechanical strength is the main constraint on its use in wound repair. It has been combined with various kinds of inorganic nanomaterials to create an efficient wound care solution to get around this limitation. It is used in a wide range of applications due to its ease of deformation into gel, nanocomposite, scaffolds, sponges, beads, powder, and film [154]. The exchange of gases between the wound and the environment plays a vital role in the healing process. Wound dressings need to allow the passage of water vapor to prevent the buildup of exudate. However, it's important to find a balance because high rates of water vapor transmission can lead to wound dehydration. Additionally, chitosan could bind to DNA, which in turn inhibits the synthesis of mRNA and interferes with the growth of microorganisms [155, 156]. Chitosan (CS) is derived from chitin and is the second most abundant polysaccharide. It has some great properties like antibacterial and hemostatic effects. The composites containing CS and cellulose were studied for their absorption of anticoagulated whole blood, antimicrobial activity, and anti-inflammatory effects. They also looked at the reduction of interleukin-6 (IL-6) and tumor necrosis factor-a (TNF-a), as well as the compatibility with human fibroblasts [157]. Chitosan has a positive charge because of its amino groups, which allows it to interact with negatively charged microbial cell membranes. This interaction disrupts the integrity of the membranes and increases permeability. As a result, the structural integrity of the microbial cells is compromised, leading to leakage of intracellular components and eventually cell death [158, 159]. Chitosan also can bind with various microorganisms like bacteria and fungi, preventing their adhesion and colonization on surfaces. It can even inhibit the activity of certain enzymes crucial for microbial survival, which adds to its antimicrobial effect. Additionally, chitosan can induce the production of reactive oxygen species within microbial cells, causing oxidative stress and further compromising their viability [160].

#### Cellulose

Cellulose, which is a biopolymer made from glucose, is widely used in various industries. While cellulose is found in both plants and bacteria, bacterial cellulose is preferred for industrial applications because of its high purity and lack of byproducts. In the context of wound healing, cellulose PCL nanofibers can be coated with chitosan and type I collagen to create chitosan collagen double layers. These layers provide support for normal fibroblast growth in human skin [161]. Cellulose derivatives have a unique chemical structure that improves their water solubility. Because of this, they can be used as a base for different formulations and have great gelation properties. These derivatives can absorb and retain exudates from the wound site, causing them to swell. As a result, the newly formulated wound dressings can effectively maintain moisture on the wound bed and allow gas exchange with the environment [162].

#### Alginate

Alginate, derived from brown marine algae, is an anionic polysaccharide that consists of β-D-mannuronic acid and β-L-guluronic acid. It forms a linear polysaccharide and is commonly used in wound treatments and dressings. Alginate dressings, particularly those with a high content of G blocks, are popular in biomedical applications because of their ease of use and low immunogenicity. These dressings work by exchanging ions like calcium and sodium, which help promote wound healing [163]. PVA/SA nanofibers containing calendula herbal extract have been used as antibacterial wound dressings in tissue engineering. Researchers have also loaded ciprofloxacin into PVA/SA nanofibers for controlled drug delivery. Growth factors including heparin-like growth factor and TGF-β1 have been loaded into PVA/alginate nanofibers to support tissue repair. Collagen-grafted PVA/SA nanofibrous has been successfully used as an artificial skin substitute. Recently, PVA/SA nanofibers blended with sodium alginate-g-N-isopropylacrylamide have shown promise as smart wound dressings for controlled delivery of diclofenac sodium, an anti-inflammatory drug [164].

#### Silk fibroin

Silk fibroin, which is produced by insects like silkworms, is a remarkable natural polymer with great potential for wound healing. It's highly biocompatible, has anti-inflammatory properties, and shows promising anti-scarring effects. Using electrospinning, silk fibroin can be transformed into nanofibers to create wound dressings with bioactive properties. These nanofibers have the potential to effectively treat burn wounds by providing a protective and healing environment. The study showed that silk fibroin was used to treat second-degree burn wounds in male Sprague–Dawley rats, resulting in significantly lower expression levels of the pro-inflammatory cytokine IL-1α compared to a gauze control treatment. This suggests that silk fibroin has anti-inflammatory properties that could contribute to promoting wound healing. The study you mentioned observed that in silk fibroin-treated wounds, the expression of TGF-β1 peaked at Day 21 post-wounding, while gauze-treated wounds peaked at Day 7. Additionally, the silk fibroin nanofibers promoted rapid collagen formation, resulting in wound healing that closely resembled normal skin rather than forming scar tissue. This suggests that silk fibroin can potentially enhance the skin's regenerative properties [165]. Natural polymers have excellent healing properties that speed up wound healing. Some biological polymers cannot be electrospuned independently because they require a lot of processing [166].

#### Collagen

Collagen, a naturally occurring protein, is renowned for its exceptional wound healing properties. As a crucial component of the extracellular matrix in tissues, collagen plays a vital role in providing structural support and facilitating cellular interactions during the wound healing process. Its inherent biocompatibility, bioresorbability, and ability to promote cell migration make collagen an ideal biomaterial for wound dressings and tissue engineering applications [167, 168]. Furthermore, collagen's unique ability to modulate various stages of wound healing, including inflammation, proliferation, and tissue remodeling, underscores its significance in promoting effective and efficient recovery of damaged tissues [24, 169].

#### Gelatin

Gelatin, derived from collagen through partial hydrolysis, possesses notable wound healing properties. This natural polymer is widely recognized for its biocompatibility, biodegradability, and versatility in various biomedical applications. Gelatin's ability to form stable gels, combined with its favorable interactions with cells and tissues, makes it an excellent candidate for wound dressings and tissue engineering scaffolds [170]. The presence of cell-binding motifs within gelatin promotes cellular adhesion and migration, contributing to tissue regeneration. Additionally, gelatin's hemostatic properties and modulation of inflammatory responses further enhance its efficacy in wound healing applications [171, 172].

#### Dextran

Dextran, a natural polymer, stands out for its notable wound healing and antimicrobial properties. Renowned for its biocompatibility and hydrophilic characteristics, dextran has garnered attention in the realm of wound care. Its ability to form films, hydrogels, or nanofibers enhances its utility in advanced wound dressings [173, 174]. Dextran's capacity to maintain moisture at the wound site creates an optimal environment for tissue repair, while its inherent antimicrobial properties contribute to preventing or managing infections. Furthermore, dextran's anti-inflammatory attributes and promotion of cell proliferation play pivotal roles in supporting the wound healing process. The multifaceted properties of dextran make it a valuable component in designing biomaterials that not only aid in the efficient recovery of damaged tissues but also provide antimicrobial benefits for enhanced wound care [175].

#### Gellan gum

Gellan gum, a natural polymer, possesses notable wound healing properties coupled with antimicrobial and anti-inflammatory attributes. Recognized for its biocompatibility and ability to form stable gels, gellan gum has emerged as a promising biomaterial in the field of wound care. Its gel-forming capabilities enable the creation of wound dressings with optimal moisture retention, promoting a conducive environment for tissue regeneration. Moreover, gellan gum exhibits inherent antimicrobial properties, contributing to infection prevention and control. The polymer's anti-inflammatory characteristics further enhance its therapeutic potential in wound healing applications by modulating the inflammatory response. Overall, gellan gum's multifaceted nature positions it as a valuable natural polymer for designing advanced wound care materials with combined wound healing, antimicrobial, and anti-inflammatory benefits [176].

#### Agarose

Agarose, a natural polymer, exhibits remarkable wound healing properties, complemented by antimicrobial and anti-inflammatory attributes, along with the capacity for nanofiber formation. Widely recognized for its biocompatibility and gel-forming abilities, agarose has found applications in advanced wound dressings and tissue engineering. Its ability to form nanofibers enhances its utility in creating biomaterials with structural similarities to the extracellular matrix, promoting cell adhesion and tissue regeneration. Additionally, agarose's inherent antimicrobial properties contribute to infection control, while its anti-inflammatory characteristics aid in modulating the immune response during the wound healing process [177, 178].

### Synthetic polymer

**Fig. 10:**
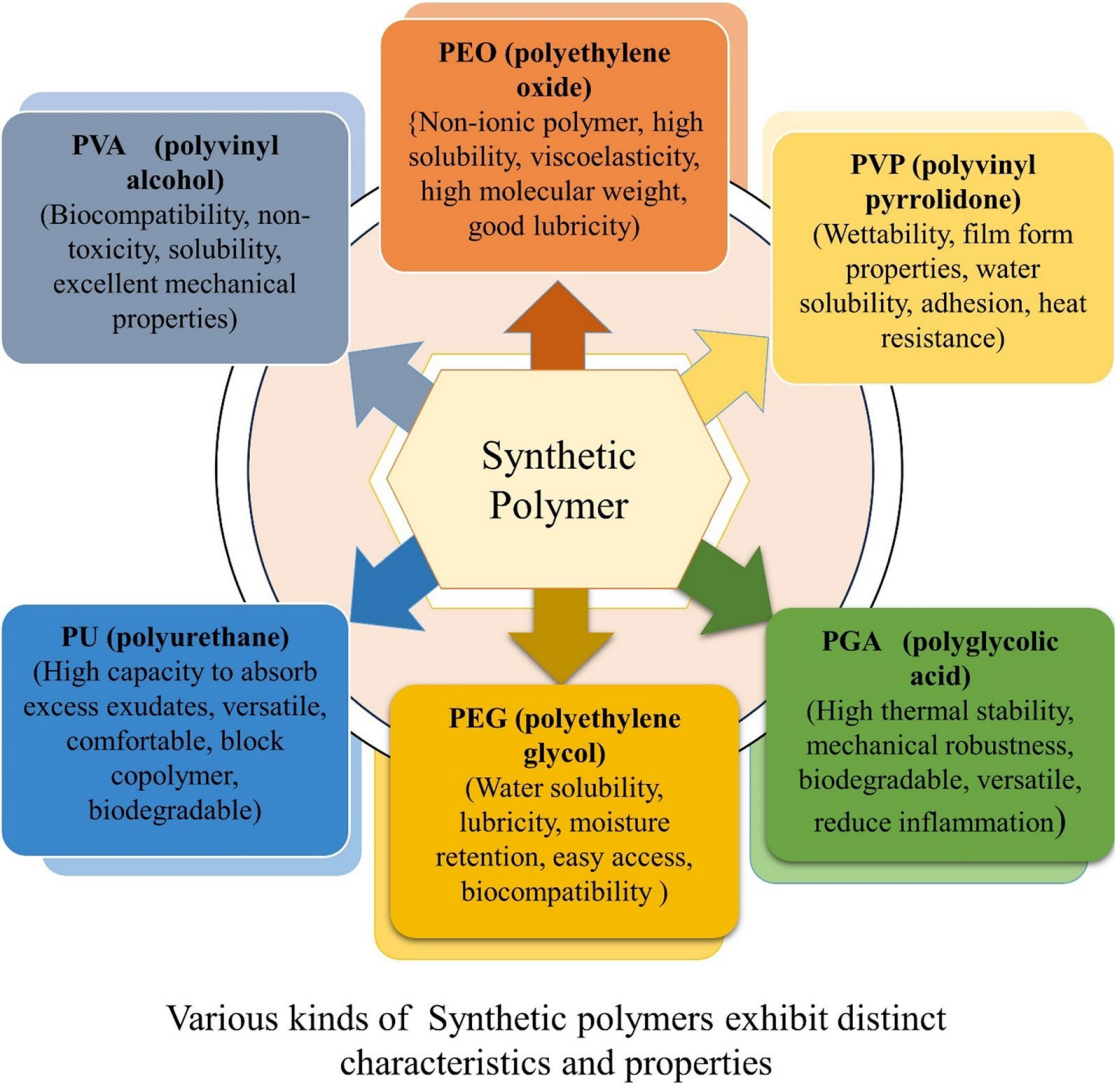
A synthetic polymer with a range of diverse polymer applications, each demonstrating unique and specific attributes related to wound healing activity

Over the years, wound dressing has undergone significant advancements. Nowadays, synthetic polymer dressings play a major role in wound management (Fig. 10). These dressings can be either passive or interactive. Passive dressings, like tulle and gauze, are non-occlusive and are used to cover wounds while promoting healing underneath **(**Table 3**)**. On the other hand, interactive dressings, which are occlusive or semi-occlusive, provide a protective barrier against bacteria [179].

#### Poly (lactic-co-glycolic acid)

Due to their distinct properties, CS and poly (lactic-co-glycolic acid) (PLGA) electrospinning presents additional difficulties. However, NFs were found to be produced by electrospinning an emulsion made by mixing CS and PVA into a PLGA solution. PVA was chosen as an emulsifier in this case and was later removed from the scaffolds. Electrospinning the remaining homogeneous emulsion, yields NFs. The mechanical test demonstrated the great mechanical strength of the framework for biomedical applications [180]. The CS/PLGA scaffold aided fibroblast attachment and proliferation, as were advantageous interactions between cells and the matrix. Consequently, CS/PLGA scaffolds produced by emulsion electrospinning are a promising candidate for wound healing [181]. One such nano-formulation is NPs, which have been extensively used in therapeutic settings. Curcumin-loaded poly (lactic-co-glycolic acid) (PLGA) NPs were prepared by emulsification solvent evaporation. The study you mentioned showed that the PLGA-curcumin nanoparticles had a slow and controlled release of curcumin over 8 days. When they were injected into the skin of mice with wounds, they found that these nanoparticles helped improve the healing of the wounds compared to using empty PLGA nanoparticles or just applying curcumin locally. Histological studies using hematoxylin–eosin and Masson's trichrome staining showed that the treatment increased collagen content, granulation tissue formation, and wound maturity. The PLGA-curcumin nanoparticles have the potential to promote tissue regeneration and improve wound healing outcomes [182]. Compared to other studies, the treatment with PLGA-curcumin nanoparticles showed a decrease in the mRNA expression of Q9GPx and NFkB. These nanoparticles have antioxidant and anti-inflammatory activity, which can benefit wound healing [183].

#### Poly (vinyl alcohol)

Poly (vinyl alcohol) (PVA) is a synthetic polymer that is not carcinogenic. It is produced by hydrolysis, alcoholysis, or aminolysis of vinyl acetate. It is utilized in various biomedical applications because of its biocompatibility, biodegradability, non-toxic, hydrophilicity, and low propensity for protein grip. It has been extensively used for drug delivery and tissue regeneration. It is a highly versatile material that can be easily shaped into different forms like fibers, particles, sponges, textiles, and films. It also has excellent hydrophilic properties and can effectively absorb fluids. Because of these characteristics, PVA, or polyvinyl alcohol, has indeed been extensively used in wound dressings for both acute and chronic wounds. It's been utilized in developing biopolymer-based dressings to improve their mechanical properties for wound healing and skin regeneration. PVA wound dressings could be particularly beneficial for treating diabetic wounds due to their strong binding affinity with glucose [184].

#### Poly caprolactone (PCL)

**Fig. 11:**
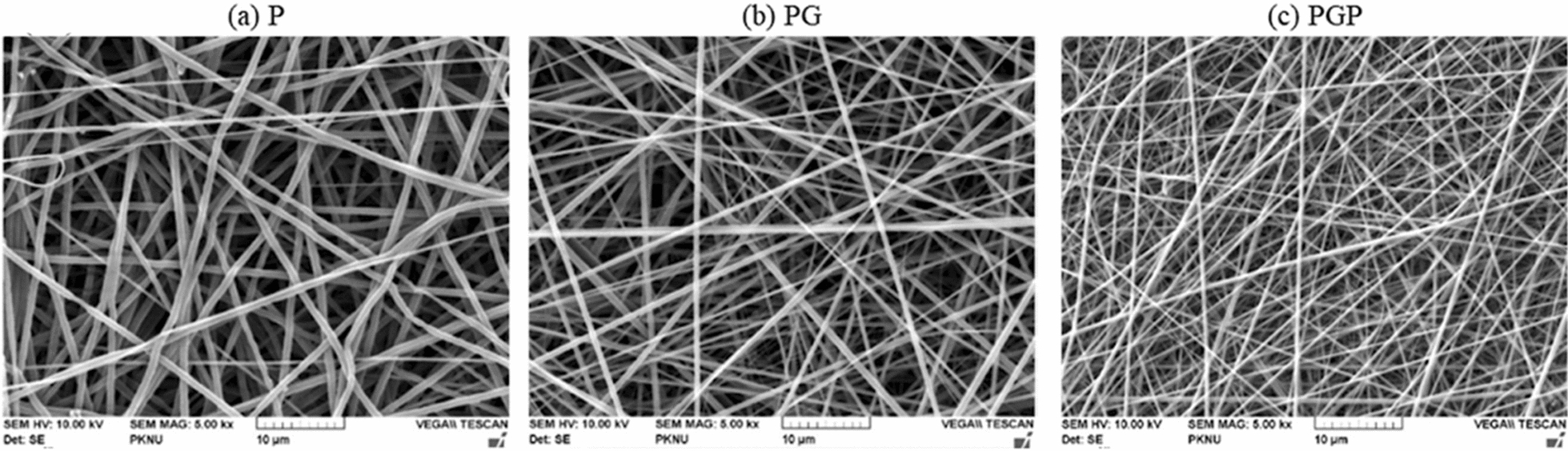
SEM images of electrospun **a** Poly-ε-Caprolactone; **b** Gelatin; **c** PCL and Gelatin NFs

A biocompatible and biodegradable polymer is produced by ring-opening polymerising caprolactone called PCL. As a medication conveyance framework supported by the FDA for use in people, PCL is broadly utilized as a biomaterial for wound mending processes as it can animate collagen creation. In other aliphatic polyesters, PCL has high hydrophobicity and slowly breaks down in the body by hydrolyzing its ester linkages. PCL is used to plan mixes with normal polysaccharides such as chitosan, alginate, or gelatin to further develop hydrophilicity, mechanical opposition, and tissue fix process applications [185]. Although synthetic polymer nanofibers can carry out structural programming, they do not have the required biological functions (Fig. 11**)**. Traditional collagen and gelatin wound dressings are often considered passive because they lack the inherent capacity to promote healing activety.

### Characteristics of nanofiber scaffold

Electrospun scaffolds have shown great potential for tissue engineering. However, their applications are still limited due to pore size and interconnectivity. These factors affect how cells infiltrate and grow into the scaffold. Using nanofibers as wound dressings, smaller pore sizes may limit cell connection and expansion on the outer layer of the nanofibers in tissue engineering applications [204]. With the growing number of wound occurrences, wound management has become critical. It has gained significant attention and is a crucial aspect of healthcare. Dressings are effective in wound management because they protect the wound's surface from infection and speed up healing. Because they encourage the migration of cells on the site of the wound as well as protect it from damage, wound dressings are leading the market for wound care. The electrospinning method is indeed used to fabricate nanofibrous dressings, which have gained widespread use in wound management. The electrospinning method is commonly used to create nanofibers with varying shapes and orientations when constructing scaffolds. These nanofibers provide a versatile and interconnected structure that is beneficial for tissue engineering and wound healing applications. The resulting non-woven scaffolds have been utilized in regenerative tissue engineering and are useful for drug delivery and treating wounds [205]. PCL (polycaprolactone) has shown great potential in developing platforms for enhancing wound healing. Nanofibers help improve biocompatibility, antibacterial properties, and the overall promotion of wound healing. The epidermal growth factor (EGF) covalently immobilizes on PCL and collagen nanofibers, it enhances the absorbency and biodegradability of the PCL nanofibers but also significantly boosts the expression of loricrin gene [206]. The human fibroblast cells could proliferate, adhere to the membrane, and maintain viability. In a nutshell, the electrospun membrane scaffold's use of synthetic and natural polymers contributed to developing wound dressings with desirable properties. Future work in wound healing includes integrating development factors, nutrients, cytokines, and other biomolecules into the platform to enhance the healing process [207]. An ideal injury dressing ought to follow the referenced qualities: (1) Control of dampness around the injury, (2) Extraordinary transmission of gases, (3) Dispose of abundant exudates, (4) Safeguard the injury from contaminations and microorganisms, (5) Decline surface corruption of wound, (6) Have mechanical security, (7) Effortlessly different and eliminated, (8) Biocompatible, biodegradable, flexible, and nontoxic, (9) Remember the injury agony, and (10) Exorbitant adequate [208].

## *In-vitro* studies

The antibacterial wound dressings stop the wound from getting infected. Bacteria and microorganisms can easily enter chronic wounds, causing inflammation and impeding the healing process for extended periods [209]. Cuts, surgeries, burns, and other wounds expose subcutaneous tissue, providing a moist, warm, and nutritious environment ideal for microorganism growth. Because they have the potential to increase trauma and put financial strain on patients, wound infections are a serious possibility. Metallic nanoparticles, like silver and gold, can interact with cellular components and generate reactive oxygen species (ROS) that can cause cellular damage. The mechanism is often associated with the toxicity observed in cancer cells, parasites, and other microorganisms. Due to their biological reactivity, silver and gold nanoparticles are known to be particularly toxic. It's crucial to prioritize the safety and compatibility of phylogenetic silver and gold nanoparticles before utilizing them in biomedical applications. In a recent study, researchers tested the toxicity of silver nanoparticles derived from *Salacia chinensis* on normal human fibroblast cells (L929) in vitro [210]. The results were promising, as the nanoparticles showed a non-toxic nature. The cell viability remained high, with over 95% viability at all the tested concentrations. The hemolysis induced was less than 3%, which falls within the biocompatible range [211]. Chitosan has strong antimicrobial properties against both Gram-positive and Gram-negative bacteria. The effectiveness of chitosan's antimicrobial properties can be influenced by factors such as the type of pathogen, the media's pH, the chitosan's structure, and its concentration. These factors play a role in determining how well chitosan can combat bacteria. The pH of the media is an essential component in the antimicrobial action of chitosan below pH 6.5, and chitosan has antimicrobial properties [212].

**Fig. 12:**
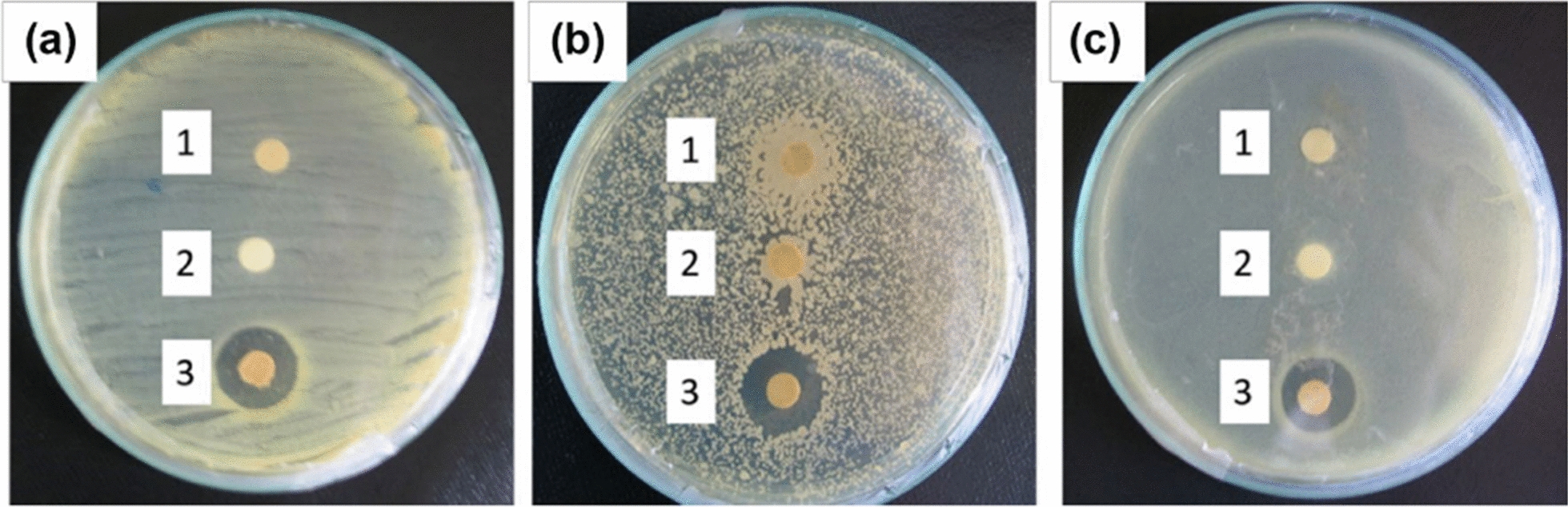
Bactericidal activity of (1) *S. spinosa* extract, (2) distilled water, and (3) biosynthesized Ag NPs from *S. spinosa* extract, against **a** *E. coli*, **b** *B. subtilis* and **c** *B. vallismortis* [124]

Recent studies have shown that a spray formulation made from the latex extract of *Jatropha curcas* has been found to possess wound-healing properties (Table 4) [213]. The formulation was observed to enhance the production of type I collagen in human fibroblasts. After just 24 h of treatment, it showed the ability to accelerate wound healing in human keratinocyte and fibroblast cells. The formulation's primary active component, curcacycline A, is a cyclic octapeptide. The formulation using *Jatropha curcas* extract exhibited antioxidant effects in the DPPH assay and displayed antibacterial properties (Fig. 12) against various bacteria typically linked to wound infections. This formulation holds tremendous promise for wound healing treatments [214]. Honey is a natural antibiotic that can effectively fight against bacterial biofilms and resistant microorganisms. Its high osmolality, acidity, and glucose oxidase content contribute to its strong antibacterial properties **(**Table 4**)**. Biofilm refers to microorganism communities in wounds, which can hinder the healing process due to increased bacterial resistance. However, honey comes to the rescue by also being effective against biofilm bacteria [215]. When curcumin was released from electrospun nanofibers made of chitosan and polylactic acid, it showed no harmful effects on L-929 fibroblast cells and increased antioxidant activity in vitro. These nanofibers significantly reduced wound size in an extraction and cut rodent wound model. In the case of electrospinning curcumin-incorporated polycaprolactone/chitosan nanofibers, about 80% of the curcumin was released within the first hundred hours [216].

**Table 4:** Synthesized plant nanoparticles and it’s biological activity

Nanoparticles	Strains	Antibacterial activity mechanism	MIC reduction %	References
CuO	*S. aureus* *P. aeruginosa*	ROS inhibition	87	[217–219]
Ag	*E. coli*	Oxidative/stress	90	[174, 175]
CoFe_2_O_4_	*E. coli* *S. aureus* *P. aeruginosa* *A. cereus*	ROS damage	60	[220]
NiFe_2_O_4_	*S. aureus* *S. pyogenes*	ROS inhibition	70	[221]
ZnO	*E. coli* *S. aureus*	ROS inhibition	99	[178, 179]
Se	*S. typhi* *E. coli*	Oxidative damage	60	[222]
TiO_2_	*E. coli* *S. aureus*	Cell Membrane damage	80	[223, 224]
CaO	*S. epidermidis* *P. aeruginosa*	Oxidative stress	80	[225]
MgO	*E. coli* *P. aeruginosa*	Cell membrane damage	70	[226, 227]
Au	*E. coli*	Oxidative and ROS damage	90	[228, 229]
Fe_2_O_3_	*L. plantarum* *L. acidophilus*	ROS inhibition	90	[230, 231]

Lipoteichoic acid, found in Gram-positive bacteria's cell walls, can stimulate the immune system and play a vital role in bacterial growth and physiology. It acts as a pathogen-associated molecular motif, which leads to nitric oxide production and activates NF-kB. This, in turn, triggers the production of pro-inflammatory mediators and cytokines [232]. Lipoteichoic acid derived from beneficial probiotics has been shown to have some amazing effects. It promotes tolerance, prevents TNF-α sepsis by inhibiting pro-inflammatory cytokine production, and enhances resistance to microbial infection in dermal cells [233].

Moreover, when applied topically, lipoteichoic acid activates toll-like receptors and human β-defensin mechanisms, providing skin protection against microbial infections and exhibiting antimicrobial properties [234]. ROS, or reactive oxygen species, play a crucial role in cellular processes like communication, differentiation, immunity, and even fighting bacteria in wounds. However, exposure to ROS for too long can lead to oxidative stress, which is harmful to cells. This oxidative stress can hinder wound-healing by causing inflammation, lipid peroxidation, DNA degradation, and enzyme inactivity. Topical application of antioxidants can help heal wounds by neutralizing free radicals and promoting skin recovery. Clinical studies have indeed shown that curcumin has antioxidant properties. In vitro experiments with a collagen network treated with curcumin demonstrated its ability to scavenge peroxide radicals. Another study using an in vivo rat model found that applying curcumin significantly reduced H_2_O_2_-induced damage to fibroblasts and keratinocytes. The use of bioactive glass-loaded nanofibers in promoting wound closure is quite promising. In a study with SD rats, those treated with these nanofibers had the smallest skin wounds by day 14, indicating their potential for promoting re-epithelialization and wound healing. Additionally, collagen, gelatin, and PHB with ostholamide have shown impressive antibacterial activity against *P. aeruginosa* [192, 193]. In recent years, numerous hemostatic dressings based on chitosan have been developed. Due to their ability to form cationic clusters that can interact with anions on red blood cells, chitosan nanofibers have remarkable hemostatic properties, accelerating platelet aggregation and ultimately stopping blood loss. This mechanism is effective even in patients with coagulation disorders and does not depend on a patient's clotting mechanism [235].

## *In-vivo* studies

**Fig. 13:**
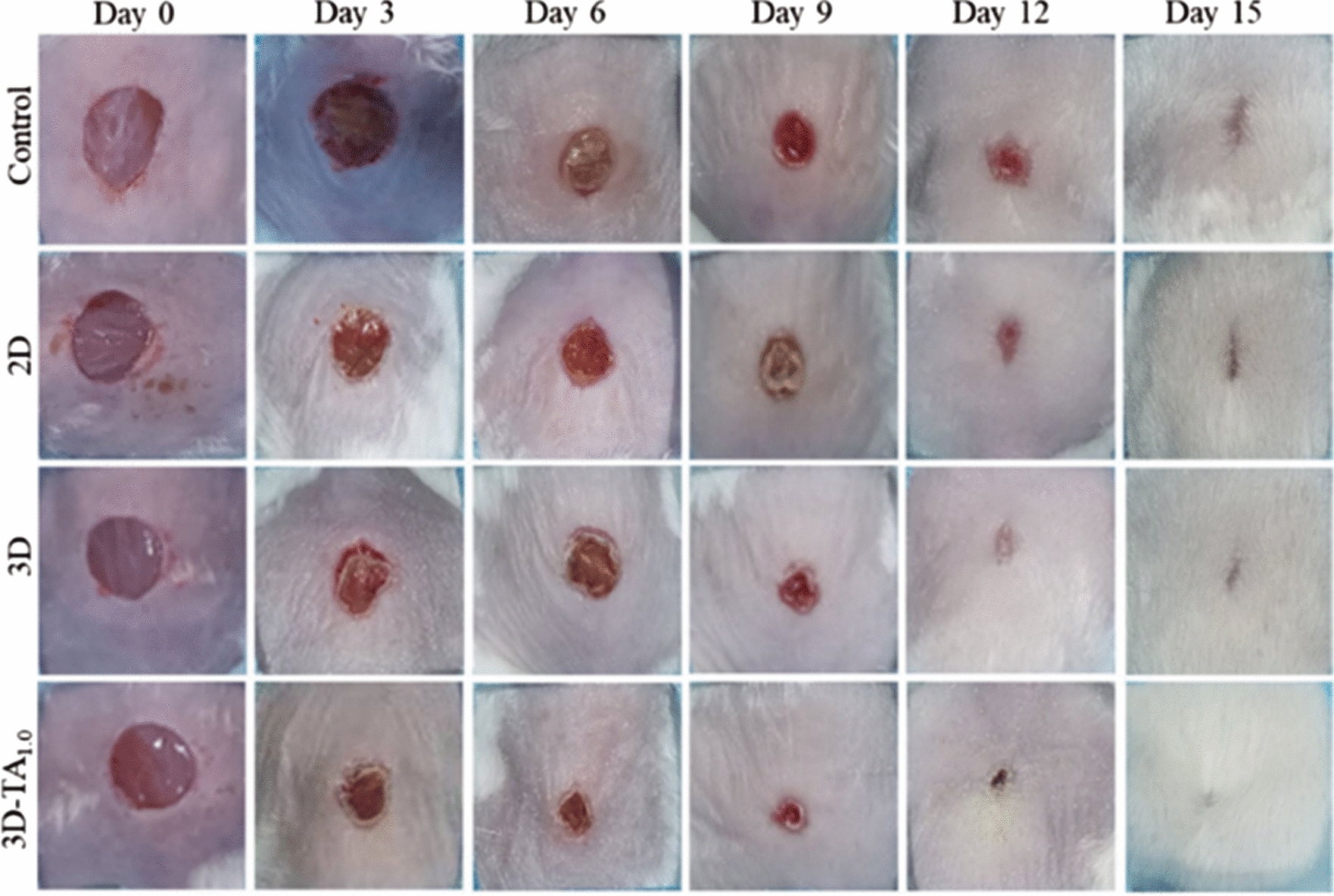
*In-vivo* healing effect **a** The photographs and wound size of full-thickness wound healing process after treating with control, 2D, 3D-TA_1.0_ at various day intervals [239]

The studies have established that rodents, especially mice, are the most commonly used animal models for wound care investigations. They have several advantages, such as their ability to reproduce quickly, allowing for multiple study generations. While higher animal models like rabbits and guinea pigs exist, porcine models, which have similar skin structure and biochemistry to humans, are less frequently employed due to cost, handling difficulties, and a lack of appropriate genetic tools [236]. Murine excisional wound models, using rats and mice, are the primary animal models used in wound healing research (Fig. 13). The anatomical, physiological, and genetic similarities between rodents and humans make them a preferred choice for various research areas. Using animal models with controlled experimental, environmental, and genetic variables allows for smaller sample sizes and more reliable results. These models will enable us to measure and monitor different stages of wound healing, especially the inflammatory and proliferative phases. Some studies tend to use male animals more frequently due to the impact of hormonal cycles in females and their heightened sensitivity to toxicity [237]. The in vivo evaluation of dual-growth factor-releasing meshes on rat skin wounds yielded promising results [238].

Meshes can be easily attached to the wounds without the need for adhesives. The electrospun meshes kept the wounds hydrated and became invisible after a few hours. The meshes with growth factors showed significantly better wound closure rates compared to other samples at weeks 1 and 2. After 4 weeks, all wounds were closed, but the 2:1 CS/PEO-NPs mesh had the smallest scar formation and more hair coverage, indicating faster healing. The research investigated how effective Pκ-CaC 8:2 and Pκ-CaC 6:4 hydrogel wound dressings were on mice with full-thickness circular excision wounds [240]. The groups treated with hydrogel showed faster wound healing than the control group, with less noticeable scarring. It's amazing how composite hydrogel dressings, like Pκ-CaC 6:4, can speed up the closure of wounds. The study also emphasized the potential of natural polymers like chitosan in promoting wound healing [241]. The study investigated the wound healing effects of ENS Mh (manuka honey) 15%@PVP mats compared to a traditional wound dressing on mice with circular wounds on their dorsal back [242]. The advanced dressing with ENS Mh@PVP showed great results in wound healing. It helped with faster epithelialization, reduced wound size, and higher-quality healing with hair growth and minimal scarring. The immunomodulatory properties of ENS Mh played a role in accelerating the healing process and promoting collagen synthesis. Manuka honey dressings also effectively prevented scarring and reduced inflammation [243].

## Perspective

Wound healing is a complex process, and traditional folk medicine has identified numerous plants with validated wound-healing properties. In vitro studies have used crude plant extracts or isolated secondary metabolites to explore their potential in wound healing further. These plants have medicinal properties for various conditions, including arthritis, snake bites, and skin diseases, and even have anticancer, anti-inflammatory, antifeedant, and muscle relaxant properties [244]. When tissue is damaged by physical, chemical, microbiological, or immunological agents, wound healing preserves the tissue's standard structure and function, ensuring its survival [245]. Electrospun nanofibers have numerous advantages for wound dressings, such as high air permeability, moisture absorption, and protection against external factors. When combined with herbal compounds, these nanofibers can further enhance their properties. It's great to see the integration of plant compounds into electrospinning techniques, as natural alternatives are in high demand due to concerns about antibiotic resistance and additives' side effects. Electro-spun fiber scaffolds will likely be multifunctional to aid in wound healing at multiple stages. Products should be designed to meet the specific needs and characteristics of different stages of wound healing. In particular, electro-spun fiber scaffolds can easily incorporate specialized stem cells, enabling a controlled and precise release of multiple drugs. This system can improve the anti-inflammatory and antibacterial activities of wounds, promote blood purification and blood separation in wounds, and ultimately promote wound healing by tailoring the amount and progress of drug release (simultaneous or continuous stepwise release) to the stage of wound healing [246]. Recent studies on electrospinning dressings in wound healing have shown great potential in enhancing the healing process. They have nanofibrous structures that mimic the extracellular matrix, controlled drug delivery systems, and even antimicrobial agents. Researchers are focusing on using biodegradable materials to make dressing removal easier, and they are also exploring ways to integrate cells within the electrospun scaffolds to promote tissue regeneration [247, 248].

Toll-like receptors (TlRs) recognize patterns in pathogens and trigger immune responses. They can detect components of protozoa, bacteria, and viruses, sending signals to the immune system. Interestingly, TlRs can also be activated by molecules released from damaged cells, known as damage-associated molecular patterns (DAMPs). These patterns are usually hidden but become recognizable during injury. TlRs and other receptors trigger inflammation when tissue damage occurs protecting tissue integrity and aiding in wound healing [249]. Gene expression regulation is a key factor in determining the quality of healing. There are significant changes in gene expression levels throughout the different stages of healing. Important genes involved in wound healing include VEGF, TGF-β, EGF, IL, and TNF-α. In the later stages of healing, EGF, TGF-β, and VEGF are upregulated, while TNF-α and IL are downregulated. In the future, we plan to study the molecular mechanisms of the healing process by quantitatively assessing the gene expression levels of these biomarkers using real-time PCR in rat skin at various stages of healing after treatment with our nanofiber [250]. While there is growing interest in using plant extracts and nanoparticles incorporated into nanofiber scaffolds for wound healing applications, there is a need for comprehensive research that assesses the effectiveness, safety, and long-term implications of such approaches in clinical settings. Many studies have shown promising results in laboratory settings, but translating these findings into safe and effective wound-healing therapies for human patients requires further investigation [251]. Research into personalized wound care takes into account an individual's genetics, microbiome, and specific wound characteristics. Optimized treatment plans based on patient-specific data to enhance the long-term healing process. Managing chronic wounds, like diabetic ulcers and pressure sores, can be quite challenging. Conventional treatments may not always be effective, leading to prolonged suffering for patients. It's crucial to explore and develop comprehensive strategies that prioritize prevention, early intervention, and long-term care for these types of wounds.

## Summary

Due to their impressive biocompatibility and wet stability, cross-linked electrospun nanofibers have shown tremendous promise in wound healing. Researchers have been diving deep into different materials and bioactive agents to enhance the healing properties of electrospun wound dressings. One particularly exciting avenue of research is the use of natural products as antimicrobial and antioxidant additives in wound dressings. By harnessing the power of nature, scientists are exploring the potential of these natural compounds to accelerate the healing process. The idea of extracting bioactive substances from various sources is also captivating. However, it's crucial to conduct thorough clinical trials to ensure the safety and efficacy of these natural products. We must address toxicity and safety concerns when incorporating them into wound dressings. Trauma dressings face diverse challenges in the future, depending on the specific type of trauma. Infection control, rapid and effective hemostasis, personalization for different wound types, biocompatibility, moisture management, long-term stability, and ease of application are all important areas of concern. The continuous advancements in wound treatment techniques, such as integrating gene editing tools, material science engineering, and interdisciplinary approaches, offer immense potential to revolutionize wound care. These innovations can enhance skin regeneration and repair, ultimately leading to more effective and efficient wound-healing methods.
